# Metformin and Glucose Concentration as Limiting Factors in Retinal Pigment Epithelial Cell Viability and Proliferation

**DOI:** 10.3390/ijms25052637

**Published:** 2024-02-24

**Authors:** Elsa Villa-Fernández, Ana Victoria García, Alejandra Fernández-Fernández, Miguel García-Villarino, Jessica Ares-Blanco, Pedro Pujante, Tomás González-Vidal, Mario F. Fraga, Edelmiro Menéndez Torre, Elias Delgado, Carmen Lambert

**Affiliations:** 1 Endocrinology, Nutrition, Diabetes and Obesity Group, Health Research Institute of the Principality of Asturias (ISPA), 33011 Oviedo, Asturias, Spain; elsavfer@gmail.com (E.V.-F.); vctrgago@gmail.com (A.V.G.); miguelvillarino@outlook.es (M.G.-V.); jessiaresb@gmail.com (J.A.-B.); pedropujanteal@gmail.com (P.P.); tomasgonvidal@gmail.com (T.G.-V.); edelangot@gmail.com (E.M.T.); delgadoelias@uniovi.es (E.D.); 2 Asturias Central University Hospital, 33011 Oviedo, Asturias, Spain; alejandraff@yahoo.es; 3 Department of Medicine, University of Oviedo, 33006 Oviedo, Asturias, Spain; 4 Nanomaterials and Nanotechnology Research Center (CINN-CSIC), Health Research Institute of Asturias (ISPA), 33011 Oviedo, Asturias, Spain; mffraga@cinn.es; 5 Centre for Biomedical Network Research on Rare Diseases (CIBERER), Instituto de Salud Carlos III, 28029 Madrid, Spain; 6 Institute of Oncology of Asturias (IUOPA), 33006 Oviedo, Asturias, Spain; 7 Department of Organisms and Systems Biology (B.O.S), University of Oviedo, 33006 Oviedo, Asturias, Spain; 8 Department of Educational Sciences, University of Oviedo, 33006 Oviedo, Asturias, Spain

**Keywords:** metformin, glucose, cell proliferation, type 2 diabetes

## Abstract

Metformin is a well-established drug for the treatment of type 2 diabetes; however, the mechanism of action has not been well described and many aspects of how it truly acts are still unknown. Moreover, regarding in vitro experiments, the glycaemic status when metformin is used is generally not considered, which, added to the suprapharmacological drug concentrations that are commonly employed in research, has resulted in gaps of its mechanism of action. The aim of this study was to determine how glucose and metformin concentrations influence cell culture. Considering that diabetic retinopathy is one of the most common complications of diabetes, a retinal pigment epithelial cell line was selected, and cell viability and proliferation rates were measured at different glucose and metformin concentrations. As expected, glucose concentration by itself positively influenced cell proliferation rates. When the metformin was considered, results were conditioned, as well, by metformin concentration. This conditioning resulted in cell death when high concentrations of metformin were used under physiological concentrations of glucose, while this did not happen when clinically relevant concentrations of metformin were used independently of glucose status. Our study shows the importance of in vitro cell growth conditions when drug effects such as metformin’s are being analysed.

## 1. Introduction

Metformin (1,1-dimethylbiguanide) is a hypoglycaemic drug that arises from traditional herbal medicine in Europe. Already in 1918, it was possible to demonstrate the effect of plants rich in this biguanide, such as *Galega officinalis*, in reducing blood glucose levels [1]. Since 1957, when Jen Sterne published the first clinical trial on the use of metformin in humans [2], metformin has been considered the first-line treatment for type 2 diabetes (T2D) due to its low toxicity, safety and efficacy in reducing glucose levels, as well as its low cost [3]. The capacity to treat T2D with an accessible and easy-to-take treatment has a grand importance considering the extent of this illness, expected to affect more than 600 million people worldwide in 2040 [4,5]. However, persistent hyperglycaemia in uncontrolled T2D can cause several complications such as cardiovascular disease, kidney failure or ocular diseases [5]. The most common and severe ocular complication is diabetic retinopathy (DR), consisting of a disorder that produces damage in the retina, eventually leading to blindness [6]. Worldwide prevalence of DR among patients with T2D is 25.1%, with an increase in the United Kingdom and Spain, with prevalences of 30.3% and 26.1%, respectively [7]. Even though DR has traditionally been classified as a microvascular disease, emerging evidence suggests that neurodegeneration can be also involved, transforming DR into a neurovascular disorder [8]. Taking into account that the most abundant cells in the retina are photoreceptors, the correlation that they can have with DR has been previously suggested [9]. Growing evidence supports this hypothesis and has determined that the release of inflammatory proteins favoured by photoreceptors can contribute to the development of DR [10,11]. Photoreceptors usually work as a unit with adjacent retinal pigment epithelium (RPE), which has also been associated with the vascular lesions characteristic of DR [12]. Hence, understanding the alterations in viability and proper function of this types of cells, as well as the reasons of induced cell death by hyperglycaemia, is a good approach in discovering new strategies to treat DR.

In addition to its use as an hypoglycaemic agent, in recent years, many other applications have been attributed to metformin, including the treatment of obesity [13], gestational diabetes [14], polycystic ovary syndrome [15] and different types of cancer, which include colorectal and ovarian cancer [16,17,18], while there is still controversy on how metformin could be beneficial in both the incidence and mortality of other types of cancer [19,20]. However, even though metformin has been used for more than 60 years [2], there are still many gaps in how exactly it carries out its effects in the body [21]. This problem supposes a limitation on its area of applicability, a result of many years of using the drug without really knowing how it exactly works.

There has been much research to establish the way of action of metformin. But, one of the major problems has been the wide variety of concentrations used both in vitro and in animal in vivo experiments [22]. Considering that metformin concentrations vary among the different organs, from ~1 mM in minor intestine to 10–40 µM or lower in circulation, it seems that metformin concentrations used in research should not be arbitrary [23]. Furthermore, not considering the effect of metformin concentration has resulted in the suggestion of mechanisms of action that cannot be seen when clinically relevant concentrations are used [24]. In vivo case-control studies and clinical trials on the use of metformin in conditions other than T2D are often conducted with the same drug concentrations of those prescribed for the treatment of T2D, ranging from 500 mg/day to up to 2500 mg/day of metformin [25,26], and mainly in T2D cohorts or without considering the glycaemic status of the patient when there is an absence of T2D. It is already known that, after ingestion, metformin begins to distribute differentially through the organs, following a route through the stomach, the intestinal tract, the liver and finally being expelled by the kidneys. Through this process, and as previously stated, metformin reaches different concentrations depending on the organ [23]. Despite this, as reviewed by LaMoia et al. [22], studies use varying concentrations of metformin, being in numerous cases suprapharmacological concentrations, and with no concerns about glucose status.

The most accepted target of metformin’s action is the liver, where metformin concentration is around 40–70 µM; there, metformin inhibits gluconeogenesis which results in a reduction in the blood levels of glucose [23]. Nevertheless, there is increasing evidence that suggests that other organs such as the gastrointestinal tract or the immune system may have an important role in metformin action [24], giving new uses to the treatment with metformin. Specifically, during the last years, different studies have highlighted the efficacy of metformin as an adjuvant in many types of tumours [27,28]. However, the need to adjust treatment doses depending on the presence of diabetes or the patient’s glycaemic levels is becoming increasingly evident [29,30]. Thus, it is generally accepted that the concentration of glucose plays a key factor in metformin action, where multiple studies in tumour cell cultures have seen that different concentrations of glucose condition cell death when exposed to metformin. The common conclusion is that glucose concentration matters and that lower glucose, more similar to blood levels than what is generally used in cell cultures, results in a higher rate of cell death when cells are exposed to equal concentrations of metformin [31,32,33].

In this study, by using an in vitro approach with a commercial human retinal pigment epithelial cell line (hTERT RPE-1), we aimed to study the influence of both metformin and glucose concentration in cell viability and proliferation.

## 2. Results

### 2.1. Influence of Glucose Media Concentration in hTERT RPE-1 Cell Viability

**Figure 1:**
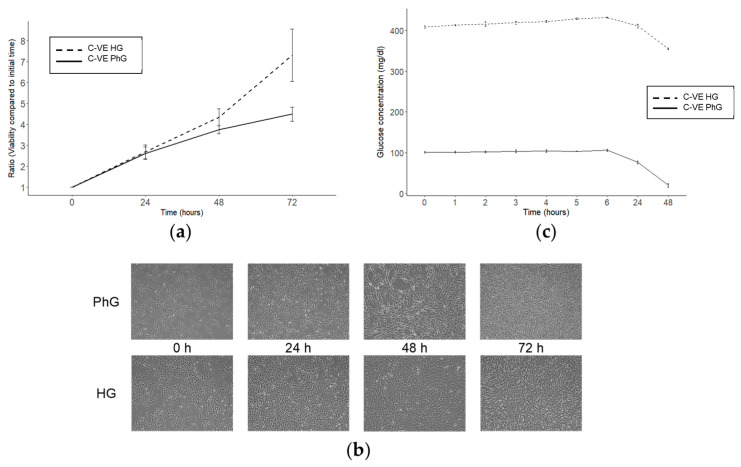
Effect of glucose concentration in the hTERT RPE-1 cell culture. (**a**) Cell viability of the hTERT RPE-1 cell line after 72 h of cell culture and under different concentrations of glucose (N = 3). (**b**) Microscopy images (magnification: 50×) of hTERT RPE-1 under different glucose concentrations. (**c**) Glucose time course for both conditions of glucose of the cell culture supernatant (N = 3). PhG: physiological glucose; HG: high glucose; C-VE: negative control.

Commonly, different cell lines are grown in cell culture with commercial media containing glucose at a concentration around 450 mg/dL, while physiological blood concentrations are between 70 and 110 mg/dL. Thus, we cultured hTERT RPE-1 cells with different glucose concentrations to analyse the influence of glucose on cell viability. We compared the cell viability, measured with a CellTiter-Blue assay, of hTERT RPE-1 cells cultured with high glucose media (HG; 450 mg/dL) and with physiological glucose media (PhG; 100 mg/dL) and observed that those cells cultured with HG media exhibit greater viability than those cultured with PhG media after 72 h of culture (Figure 1a,b).

Then, we decided to measure the glucose media concentration for 48 h of the cell culture, and observed that after this time, glucose in the PhG media was totally consumed, while in the HG media, glucose concentrations, although being reduced, remained higher than 300 mg/dL (Figure 1c).

### 2.2. Metformin Concentration Affects Growth in a Glucose-Dependent Manner

**Figure 2:**
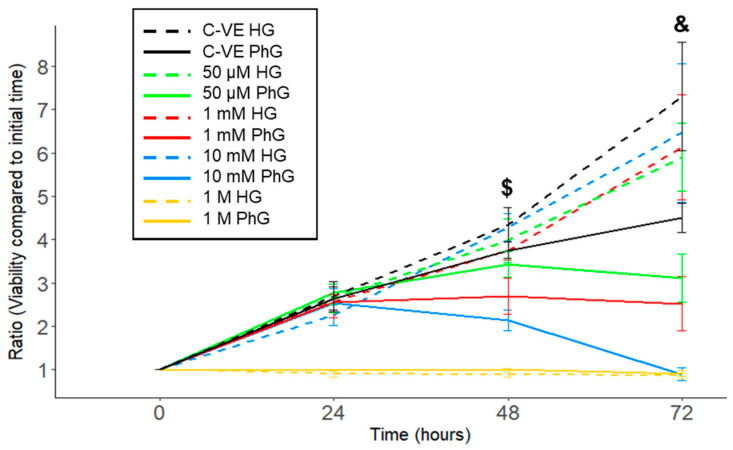
hTERT RPE-1 cell viability time course comparing the different concentrations of metformin (1 M, 10 mM,1 mM, 50 µM) under both conditions of glucose (N = 3). PhG: physiological glucose; HG: high glucose; C-VE: negative control. ($: *p* = 0.020; &: *p* = 0.012; 10 mM PhG vs. 10 mM HG).

In order to analyse the effect of metformin concentration in the cell cultures, a CellTiter-Blue assay was used to analyse the viability of cells grown in HG and PhG media and with different metformin concentrations (1 M, 10 mM, 1 mM, 50 µM and 0 µM) and cultured for 3 days. We observed that cell viability was inversely correlated with metformin concentration, independently of glucose media concentration, although a high glucose media concentration protected cells from dying under the different metformin conditions (Figure 2, Table S1).

Specifically, 1 M metformin concentration induced cell death after 12 h of culture in both glucose media concentration groups, while with the 10 mM metformin concentration, cells supplemented with PhG media started to die after 48 h of cell culture, whereas in the HG media, cells remained alive (*p* = 0.020), with an increased difference when reaching 72 h (*p* = 0.012). In the case of the 1 mM and 50 μM metformin concentrations, cell viability was increased in those cells harvested with HG media compared to PhG media, which was also higher the lower the metformin concentration.

### 2.3. Influence of Glucose and Metformin Concentration on Cell Proliferation Rate

**Figure 3:**
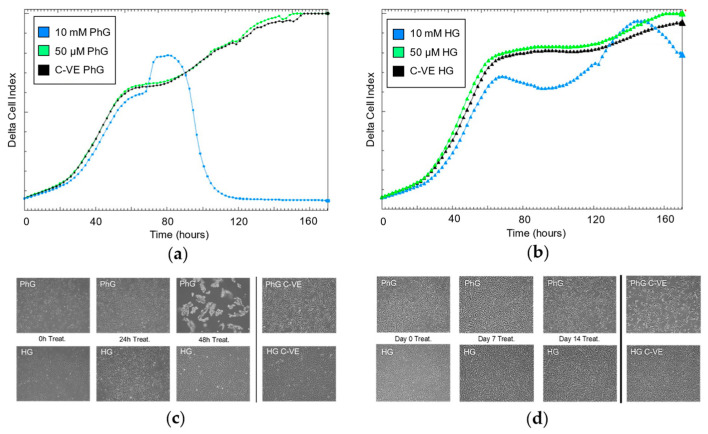
Continuous cell proliferation rate registry by iCelligence under two concentrations of metformin (10 mM, 50 µM) (**a**) and physiological (**b**) and high concentrations of glucose (N = 3). Cell culture of hTERT RPE-1 (magnification: 50×) under the two concentrations of glucose and for (**c**) 10 mM of metformin and (**d**) 50 µM of metformin. PhG: physiological glucose; HG: high glucose; C-VE: negative control.

As previously seen, the effect on cell proliferation rate under different metformin concentrations is conditioned by glucose concentration. Specifically, regarding the 50 µM metformin concentration, after 48 h of cell culture, there was no glucose conditional effect on how the treatment affects cell growth. By contrast, for the 10 mM metformin concentration, glucose concentration is a key factor in cell growth, where under physiological glucose conditions, the proliferation rates plummeted after 80 h, while the high glucose conditions enabled cells to continue growing even after a long exposure with a high concentration of metformin (Figure 3a,b). These results are concordant with what we observed by microscopy of the hTERT RPE-1 cell culture, where cells treated with the high metformin (10 mM) and PhG concentration started to die after 48 h of culture (Figure 3c); however, when exposed to the HG concentration, cells remained alive for the same period. On the contrary, low metformin concentration (50 µM) did not induce cell death under both concentrations of glucose even after 14 days of cell culture (Figure 3d).

### 2.4. Metformin Concentration Influences Glucose Consumption in Cell Cultures

**Figure 4:**
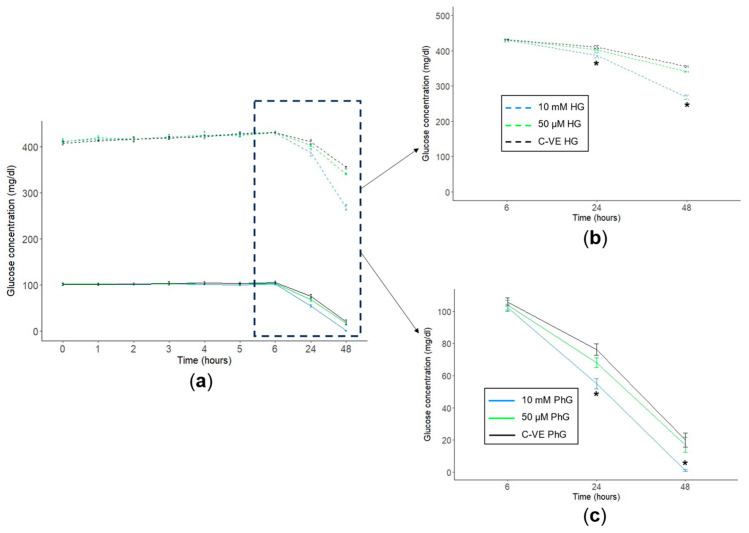
Glucose consumption time course. (**a**) Cell supernatant glucose consumption time course for the two concentrations of glucose. Cell supernatant glucose concentration at 6 h, 24 h and 48 h of (**b**) high and (**c**) physiological concentration culture media for the different concentrations of metformin (C-VE, 10 mM, 50 µM) (N = 3). PhG: physiological glucose; HG: high glucose; C-VE: negative control. * *p* < 0.05 (10 mM vs. C-VE).

Cell supernatant was obtained from hTERT RPE-1 cells treated with different glucose (PhG and HG) and metformin (10 mM and 50 µM) concentrations, where glucose concentration was measured for 48 h. Regarding the metformin concentration, after 48 h of culture, those cells treated with suprapharmacological concentrations of metformin (10 mM) consumed a higher amount of glucose than those treated without metformin or with 50 µM concentrations (Figure 4a). Specifically, in cells cultured with HG media, glucose concentration was significantly reduced from 355 mg/dL in the control group to 268 mg/dL in the 10 mM treatment group, while glucose concentration in the pharmacological group (50 µM) was similar to the control group (341 mg/dL) (Figure 4b). Considering those cells cultured in physiological glucose conditions, cells treated with metformin 10 mM almost halved glucose concentration after 24 h of culture (54.9 mg/dL), being nearly consumed after 48 h of culture (1.07 mg/dL). By contrast, regarding the control group and the pharmacological treatment conditions (50 µM), the glucose concentration after 24 h of cell culture was 76.2 mg/dL and 68.0 mg/dL, respectively, and after 48 h, it dropped to 20.0 mg/dL and 17.0 mg/dL, respectively, as well (Figure 4c).

## 3. Discussion

Patients with high blood glucose levels have a high risk of developing damage in their blood vessels. When these blood vessels are located in the retina, this results in the development of DR, which can lead to blindness if remained untreated [5,34]. Among the structures that become damaged in DR, we can find the photoreceptors and the RPE. These cells play a crucial role in maintaining proper visual function but have recently been associated with the development of DR by the release of inflammatory proteins that favour the development of vascular lesions [35]. In addition, to better understand the correlation between photoreceptors–RPE and DR, it is necessary to remark that the retina is a highly energy-demanding structure which needs a steady supply of glucose [36], which is provided via the RPE [37].

Therefore, using an RPE in vitro model to study glucose and metformin effects seems coherent to assess the importance of both glucose and metformin concentrations in cell proliferation and viability. Here, we show how the selected growth conditions can influence a response in the hTERT RPE-1 cell line, particularly for different conditions of glucose and metformin. Similar results have also been seen in other studies when using metformin to induce cell death in cancer cells [32,38] as well as changes in glucose concentration condition cell response [33]. Using different concentrations of metformin, from clinically achievable to suprapharmacological concentrations, and exposure to two different concentrations of glucose in the growth media resulted, indeed, in changes in cell proliferation and viability. For the glucose concentration, even though there were not significant differences, a tendency could be seen where viability was reduced in PhG (100 mg/dL) media compared to HG (450 mg/dL), suggesting that glucose by itself has a limiting effect in this cell line. Moreover, the maximum confluency was reached before in the cells exposed to HG than PhG.

For the metformin treatment, a range of concentrations was used to analyse the metformin concentration effect when applying possible clinically relevant concentrations (50 µM,1 mM), metformin concentrations that have been extensively used in in vitro studies (10 mM) and, finally, a high suprapharmacological concentration of metformin (1 M). The consideration of concentrations of metformin that can be obtained in vivo makes results have a more clinical value, compared with previous studies where only high concentrations of metformin were analysed [31,32,33,39]. To see the effect of these metformin concentrations, the viability of cells was measured based on the reduction process of resazurin to resorufin. It could be observed that compared to the control, cell viability was lower the higher the metformin concentration, showing that, in fact, metformin concentration is not arbitrary and can affect cell life expectancy. Further, when glucose concentration was also considered, there was an additional cell effect that can be clearly seen for the 10 mM metformin concentration, where cells exposed to PhG suffered total cell death after 72 h, probably due to total glucose consumption achieved at 48 h of treatment. On the contrary, when exposed to HG, even after 72 h, glucose in media was still present, and cells did not show major cell death. This gives rise to the problem that most standard growth medias are formulated based on a high glucose content to avoid nutrient deprivation over time; nonetheless, the possibility that high glucose levels may mask true drug responses is not really considered [31,40].

Nowadays, there is an advocacy for using metformin for more than just treating T2D, such as for cancer treatment, polycystic ovary syndrome and the treatment of obesity, among others [41,42]. However, despite metformin’s proven safety throughout the years, especially in T2D, there are still many missing answers on how metformin works, which organs it affects and how the concentration of not only metformin but glucose can affect how metformin acts. To reach that point, there should be a common agreement on how both in vitro and in vivo assays should be performed for the results to be comparable, as it has been seen that just by changing the cell line that is used, the effect of metformin changes [31,43].

Due to repurposing metformin for cancer treatment [29,44], a large part of the research conducted in vitro with metformin is performed with cancer cell lines. But, considering that cancer cells do not behave in the same way as noncancerous cells, especially in the mechanisms related to energy metabolism [45], the results obtained can be conditioned. Moreover, due to hepatic gluconeogenesis inhibition being the most generally accepted mechanism of metformin action [23], a substantial part of the research conducted with metformin has been carried out with hepatic in vitro and in vivo models [22]. This leaves a gap in the metformin action of other cellular types, supporting the selection of the RPE cell line used for this study.

Therefore, even though metformin is an established drug for the treatment of T2D, there is still gaps in many aspects of how it exactly works and which metabolic routes it affects, which is of great importance when it comes to using this medication for the treatment of other diseases. But, before investigating that, the establishment of the standardized concentration of metformin that should be used in research is a key factor in determining the grounding of metformin action. Otherwise, as has already happened with a vast number of studies, the results will not have clinical relevance. Following this, we have proved here that it is important to determine the concentration of glucose and metformin that should be used considering how metformin distributes in the different organs and that the concentration of metformin varies throughout the body [21,46], as well as that results are conditioned by glucose cell status. Therefore, considering that some types of cells consume more glucose (e.g., retinal cells) [36,47] or that cancer cells have a higher energy metabolism [48], our findings suggest that it is important to consider patients’ glucose status when metformin is used, and that metformin could be used for more than treating T2D, such as cancer treatment, if we were able to administer not-harmful high doses of metformin accompanied by a low concentration of glucose.

Additionally, the results can also have relevance in DR therapy, an emerging issue where in vitro studies have found metformin to reduce cellular stress events [49] and demonstrate a neuroprotective action of metformin in in vivo animal models [50,51]. However, the use of high concentrations of metformin, compared to plasma concentrations [21,52], in research is still spread. Further, if high metformin concentrations were to be used in a clinical context, they could lead to the opposite effect that was meant to be obtained, which could favour metformin-associated toxicity and, sporadically, blindness [53,54].

Metformin has been used for years for the treatment of T2D; however, in recent years, its use has been extended to a multitude of different pathologies, highlighting its use in oncological diseases. Our results highlight the influence of glucose and metformin concentrations on a retinal pigment epithelial cell line (hTERT RPE-1). These findings show that glucose and metformin concentrations have a direct influence on the effect of metformin on cells.

According to these results, it is important to highlight the need to carry out, for each pathology and cell line analysed, the optimal and physiological conditions of the glucose and metformin dose treatment, and that this is just an example of how, in a pathology as well-known as diabetic retinopathy, where we know that glucose levels are usually higher than those of the nondiabetic population, the choice of a high dose of the drug causes a reduction in cell viability, and even cell death, which, although it may be beneficial in pathologies such as cancer, can have a very harmful effect on the other target organs of metformin. Therefore, although the results described here have great clinical relevance, establishing the need to adjust the treatment dose depending on the patient’s glycaemic status, it is necessary to carry out an in-depth analysis of the mechanisms of action of treatment with metformin in the different pathologies for which it can be used.

### Limitation of the Study

Although these results make clear the importance of adjusting the doses of the metformin treatment based on the glucose levels in the culture medium in the hTERT RPE-1 cell line, related to diabetic retinopathy, it would be necessary to perform an independent cell viability analysis prior to carrying out any work, using in each case the corresponding cell line, as well as the expected glucose concentration, and never generalizing the conditions set out here, which are only valid for the RPE-1 cell line.

## 4. Materials and Methods

### 4.1. Cell Line

In order to select the most appropriate cell line to assess the influence of glucose media concentration in the proliferative behaviour of different cell lines, five different cell lines were cultured under different glucose concentrations and treated with metformin 10 mM (See Supplementary Materials and Figure S1). Finally, the hTERT-immortalized retinal pigment epithelial cell line (hTERT RPE-1) was used in this study. Cells were obtained from the American Type Culture Collection (ATCC), cultured under humidified atmosphere in a 5% CO_2_ incubator at 37 °C, and the culture medium used was DMEM F-12 (Dulbecco’s Modified Eagle Medium: Nutrient Mixture F12, Gibco) supplemented with 10% foetal bovine serum (FBS), penicillin (100 U/mL) and streptomycin (100 μg/mL).

### 4.2. Cell Viability Assays

A CellTiter-Blue assay (Promega, Fitchburg, WI, USA) was used for the cell viability measurement. Briefly, hTERT RPE-1 cells were seeded in quadruplicate for the different glucose and metformin concentrations in four different 96-well microplates at a density of 5 × 10^3^ in 100 μL of complete DMEM F-12 medium and cultured overnight. Once the cells had adhered to the plate surface, cells were treated with varying concentrations of glucose, complete DMEM supplemented with a physiological concentration of glucose (PhG: 100 mg/dL; Gibco, New York, NY, USA, EEUU) or complete DMEM supplemented with high glucose concentrations (HG: 450 mg/dL; Gibco, New York, NY, USA, EEUU), and metformin, 1 M, 10 mM, 1 mM and 50 μM (Cayman Chemical Company, Ann Arbor, MI, USA, EEUU), and cultured for 0 h, 24 h, 48 h and 72 h. At last, 20 μL of the CellTiter-Blue reagent was added to each well at different times and incubated at 37 °C for 1 h. Then, the absorbance was measured at a wavelength of 590 nm using a Synergy|HT Biotek reader (Biotek Instruments, Winooski, VT, USA) and the All-In-One Microplate Reader Software-Gen 5 version 2.00.18 (Biotek Instruments, Winooski, VT, USA).

### 4.3. Cell Cultures

Additionally, the cells were seeded in P100 and P150 plaques under 50 µM and 10 mM metformin treatments, respectively. For the P100 plaques, the cells were seeded at a density of 2 × 10^5^ with DMEM F-12 during 24 h, after which, the cells were cultured during 24 h with DMEM without FBS for cell arrest. After this time, the medium was changed to complete DMEM, and the cells were cultured for 14 days. For the P150 plaques, the workflow was the same except that 6 × 10^5^ cells were seeded and that the culture with complete DMEM was carried out during just 48 h. For both assays, the cells were cultured parallelly under the two concentrations of glucose (PhG and HG).

### 4.4. Proliferation Analysis

Cell proliferation rates were measured using an iCELLigence Real Time Cell Analyser (RTCA, ACEA Biosciences, San Diego, CA, USA). Triplicates of hTERT RPE-1 cells were seeded onto analyser specific plates. Two metformin (10 mM and 50 μM) and two glucose (PhG and HG) concentrations were simultaneously used and compared. Cell impedance was measured every 2 h for 8 consecutive days through microelectric biosensors located at the base of the plate wells. Cell proliferation was represented by Delta Cell Index (DCI (t) = CI(t) − CI (tdelta) + Delta Constant) and doubling time parameters.

### 4.5. Glucose Concentration

Using cell supernatant, the photometric detection of glucose was performed by using the GLUC3 in vitro test (cobas^®^, Roche diagnostics, Mannheim, Germany) in the biochemical service of the Hospital Universitario Central de Asturias (HUCA, Oviedo, Asturias, Spain). Briefly, glucose is phosphorylated by adenosine triphosphate (ATP) in the reaction catalysed by hexokinase. Glucose-6-phosphate (G6P) is then oxidized to 6-phospho-gluconate in the presence of oxidized nicotinamide adenine dinucleotide (NAD) in a reaction catalysed by glucose-6-phosphate dehydrogenase (G6PDH). During this oxidation, an equimolar amount of NAD is reduced to NADH. The consequent increase in absorbance at 340 nm is directly proportional to glucose concentration.

### 4.6. Statistics

The analysis was performed using the JASP version 0.17.1.0 statistical software. A nonparametric Kruskal–Wallis test followed by a Dunn’s post hoc test was performed. A *p*-value ≤ 0.05 was used to define associations as statistically significant. The number of replicates (N) are given in the figure legends.

## Data Availability

The datasets generated and/or analysed during the current study are available upon request.

## Supplementary Materials

The supporting information can be downloaded at https://www.mdpi.com/article/10.3390/ijms25052637/s1.
