# Supplementary material for: Metformin and Glucose Concentration as Limiting Factors in Retinal Pigment Epithelial Cell Viability and Proliferation
Source: Int J Mol Sci. 2024 Feb 24;25(5):2637. doi: 10.3390/ijms25052637 (PMC10932269; doi:10.3390/ijms25052637)
Supplement: Supplementary file 1 [file ijms-25-02637-s001.zip › ijms-2856734-supplementary.pdf]

Supplementary Material

Metformin and Glucose Concentration as Limiting Factors in Retinal Pigment Epithelial Cell Viability and Proliferation

Elsa Villa-Fernández [1,†], Ana Victoria García [1,†], Alejandra Fernández-Fernández [2], Miguel García-Villarino [1,3], Jessica Ares-Blanco [1,2,3], Pedro Pujante [1,2], Tomás González-Vidal [1,2], Mario F. Fraga [4,5,6,7], Edelmiro Menéndez Torre [1,2,3,5], Elias Delgado [1,2,3,5,‡] and Carmen Lambert [1,8,*,‡]

[1] Endocrinology, Nutrition, Diabetes and Obesity Group, Health Research Institute of the Principality of Asturias (ISPA), 33011 Oviedo, Asturias, Spain; elsavfer@gmail.com (E.V.-F.); vctrgago@gmail.com (A.V.G.); miguelvillarino@outlook.es (M.G.-V.); jessiaresb@gmail.com (J.A.-B.); pedropujanteal@gmail.com (P.P.); tomasgonvidal@gmail.com (T.G.-V.); edelangot@gmail.com (E.M.T.); delgadoelias@uniovi.es (E.D.)

[2] Asturias Central University Hospital, 33011 Oviedo, Asturias, Spain; alejandraff@yahoo.es

[3] Department of Medicine, University of Oviedo, 33006 Oviedo, Asturias, Spain

[4] Nanomaterials and Nanotechnology Research Center (CINN-CSIC), Health Research Institute of Asturias (ISPA), 33011 Oviedo, Asturias, Spain; mffraga@cinn.es

[5] Centre for Biomedical Network Research on Rare Diseases (CIBERER), Instituto de Salud Carlos III, 28029 Madrid, Spain

[6] Institute of Oncology of Asturias (IUOPA), 33006 Oviedo, Asturias, Spain

[7] Department of Organisms and Systems Biology (B.O.S), University of Oviedo, 33006 Oviedo, Asturias, Spain

[8] Department of Educational Sciences, University of Oviedo, 33006 Oviedo, Asturias, Spain

* Correspondence: carmen.lambert@ispasturias.es

† These authors contributed equally to this work.

‡ These authors contributed equally to this work.

Table S1. hTERT RPE-1 cell viability time course measured with Cell Titter Blue assay

		N=1		N=2		N=3		MEAN	
		**PhG**	**HG**	**PhG**	**HG**	**PhG**	**HG**	**PhG**	**HG**
**0h**	DMEM	1.000	1.000	1.000	1.000	1.000	1.000	1.000	1.000
	Control	1.260	1.162	1.119	1.159	1.164	1.093	1.181	1.138
	1M	1.210	1.473	1.131	1.173	1.194	1.156	1.178	1.268
	10mM	1.255	1.226	1.133	1.100	1.167	1.199	1.185	1.175
	1mM	1.309	1.305	1.172	1.292	1.273	1.186	1.251	1.261
	50µM	1.233	1.333	1.083	1.329	1.342	1.206	1.219	1.289
**24h**	DMEM	1.000	1.000	1.000	1.000	1.000	1.000	1.000	1.000
	Control	4.053	3.988	2.804	3.921	2.934	2.354	3.264	3.421
	1M	1.198	1.248	1.239	1.218	1.237	1.254	1.225	1.240
	10mM	3.900	3.347	2.459	3.204	3.107	2.242	3.156	2.931
	1mM	3.844	4.006	3.209	4.675	3.061	2.265	3.372	3.648
	50µM	4.062	4.713	3.251	4.122	3.287	2.602	3.533	3.812
**48h**	DMEM	1.000	1.000	1.000	1.000	1.000	1.000	1.000	1.000
	Control	5.102	6.754	4.415	5.259	4.719	4.960	4.745	5.658
	1M	1.210	1.294	1.299	1.272	1.344	1.261	1.285	1.276
	10mM	3.251	6.817	2.471	5.492	2.408	5.018	2.710	5.776
	1mM	2.441	6.081	4.010	5.185	4.471	4.906	3.641	5.391
	50µM	4.909	7.409	4.051	4.969	4.424	5.202	4.461	5.860
**72h**	DMEM	1.000	1.000	1.000	1.000	1.000	1.000	1.000	1.000
	Control	5.388	7.544	7.057	13.817	6.368	8.549	6.271	9.970
	1M	1.054	1.273	1.381	1.268	1.398	1.320	1.278	1.287
	10mM	0.942	6.261	1.192	12.957	1.617	7.678	1.250	8.966
	1mM	5.485	6.797	2.959	13.448	2.713	7.604	3.719	9.283
	50µM	5.647	7.251	4.160	11.941	3.473	8.143	4.426	9.112

Absorbance measurements are normalized against DMEM (cultured in the same conditions but without cells).Absorbance measurements from triplicates at a wavelength of 590 nm

Supplementary methods

Cell line selection

In order to select the most appropriate cell line to assess the influence of glucose media concentration in the proliferative behaviour of different cell lines, five different cell lines were selected: hTERT RPE-1 (retinal epithelial cell line), MRC-5 (lung cell line), THLE-2 (liver cell line), MCF7 (breast cell line) and A2780 (ovarian cell line). Cells were treated with metformin and under two different glucose media concentration: high glucose media (HG; 450 mg/dl), and with physiological glucose media (PhG; 100 mg/dl). Metformin treatment concentration was stablished at 10 mM due to it being the most common metformin concentration used on literature. We could observe with microscopy images that treatment with 10mM metformin causes morphological changes under PhG conditions. Cells lose adherence to the plate, become rounded and decrease in size but continue to retain their refractive properties. On the contrary, under HG conditions the cells maintain a morphology like the control (Figure **S1**). Finally, only the hTERT RPE-1 cell line was selected to carry out all viability and proliferation analyses due to its greater relationship with diabetic pathology and treatment with metformin.

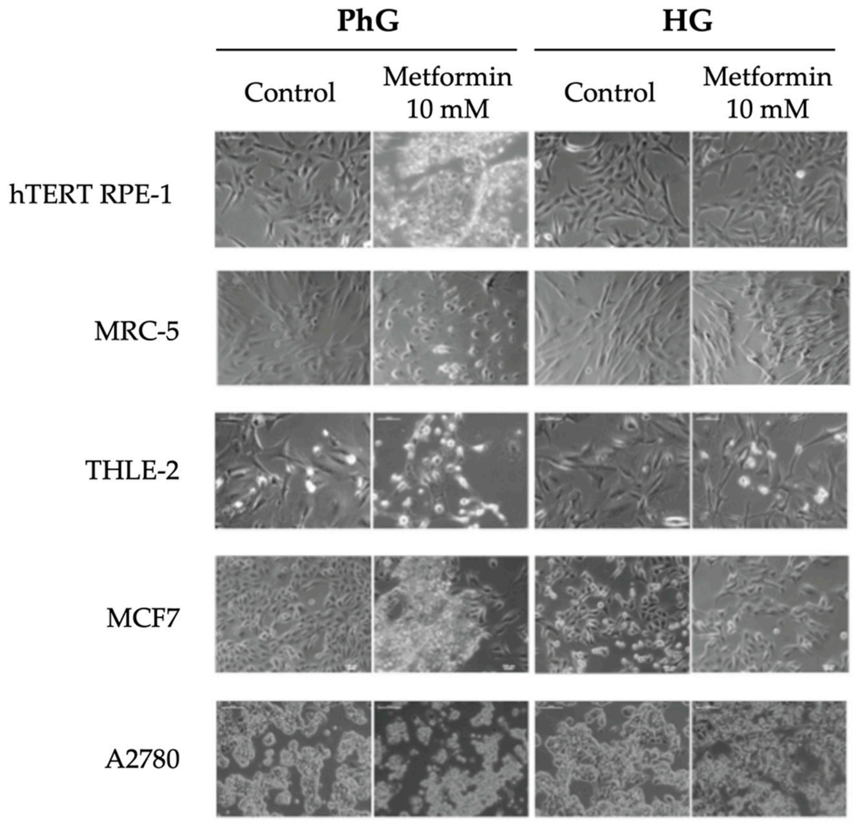

Figure S1. *Cell culture (Magnification 10×) of different cell lines under two different glucose concentrations comparing control to treatment with 10 mM metformin. PhG: physiological glucose; HG: high glucose*
